# Monitoring Progress in Equality for the Sustainable Development Goals: A Case Study of Meeting Demand for Family Planning

**DOI:** 10.9745/GHSP-D-18-00012

**Published:** 2018-06-27

**Authors:** Yoonjoung Choi, Madeleine Short Fabic

**Affiliations:** a Department of Population, Family, and Reproductive Health, Bloomberg School of Public Health, Johns Hopkins University, Baltimore, MD, USA.; b Office of Population and Reproductive Health, Bureau for Global Health, U.S. Agency for International Development, Washington, DC, USA.

## Abstract

**Objectives:**

Equality is a central component of the Sustainable Development Goals (SDGs). We took one SDG indicator and benchmark—percent of family planning demand met with modern contraceptives, with a benchmark of at least 75% in all countries—as a case study to illuminate recommendations for monitoring equality. Specifically, we assessed levels, patterns, and trends in disparity by key background characteristics and identified disparity measures that are programmatically relevant and easy to interpret.

**Methods:**

Data were from the Demographic and Health Surveys in 55 countries that have conducted at least 2 surveys since 1990. We calculated absolute difference among subgroups, disaggregated by age, education, household wealth quintile, urban/rural residence, subnational region/administrative unit, and marital status. Our unit of analysis was survey, and we conducted largely descriptive analyses. To understand trends in disparity, we used a fixed-effect linear regression model to estimate an annual rate of change in absolute differences.

**Results:**

A significant level of disparity existed across various background characteristics, ranging from a median difference of 5 percentage points by marital status to 32 percentage points by administrative unit. On average across the study countries, national level of met demand has increased over time while disparity has declined in most disaggregates including by education, wealth, residence, and age. We found statistically significant positive correlations among 4 disparity measures—education, wealth, residence, and administrative unit. Disparities by wealth quintile were easiest to interpret over time and across countries.

**Conclusions:**

At the global level, we recommend monitoring disparity in met demand by wealth quintile, which is strongly correlated with disparity by education, residence, and region and comparable across countries and over time. For monitoring by individual countries and for programmatic purposes, we further recommend monitoring disparity by first-level administrative unit, which can provide direct programmatic relevance.

## Abstract (precis)

As demand for family planning has increasingly been satisfied, disparities between groups within a country have also generally declined but persist. To monitor disparity across countries and over time, we recommend comparing met demand by wealth quintile because it is most comparable to interpret and highly correlated with disparity by education, residence, and region. Within country, comparing disparity in met demand across geographic region can identify populations with greater need for programmatic purposes.

## BACKGROUND

The Sustainable Development Goals (SDGs) are a set of 17 goals in support of people, planet, and prosperity for every country to achieve by 2030. Each goal has specific targets and each target is monitored through at least 1 indicator, identified and defined in the United Nations Global Indicator Framework for the SDGs.^1^ With 193 participating countries, 17 goals, and 169 targets, SDG indicators are parsimonious per target; even so, the Global Indicator Framework recommends a total of 232 indicators to monitor progress in each country and globally.

Adding to the complexity and importance of SDGs monitoring is the need to monitor inclusiveness and equality.^2^^–^^4^ In response, the SDGs have an overarching principle of data disaggregation^5^:

*indicators should be disaggregated, where relevant, by income, sex, age, race, ethnicity, migratory status, disability and geographic location, or other characteristics, in accordance with the Fundamental Principles of Official Statistics.*

Disaggregates will vary based on country context. For example, in ethnically homogeneous countries, geographic information may be more important than ethnic disaggregates. Such differences in country context present challenges for monitoring disparity at the global level,^6^ where data must be available and comparable to inform global estimates, projections, and focus. Additionally, with such a large set of indicators, disaggregation must be as prudent as possible to facilitate measurement and accountability.

SDG Goal 3 is devoted to health and one of its targets, target 3.7, is specific to reproductive health^2^:

*By 2030, ensure universal access to sexual and reproductive health-care services, including for family planning, information and education, and the integration of reproductive health into national strategies and programmes.*

Progress in achieving the family planning component of the target is monitored with the indicator, “demand for family planning met with modern contraception” (hereinafter referred to as “met demand”), with a proposed benchmark of “at least 75 percent in all countries by 2030.”^7^^–^^9^ The above-mentioned disparity measurement challenges apply to the met demand indicator. A critical question is what disaggregates should be used to monitor subnational disparity, both within a given country and across countries?

Progress in achieving the family planning SDGs target is monitored by assessing demand for family planning met with modern contraception.

Our article aims to address the data disaggregation challenge using the SDG indicator of reproductive health—met demand—to explore various measures of disparity and their trends, and to make recommendations about appropriate global-level disaggregates. Specific aims are to: (1) assess levels, patterns, and trends in disparity for met demand by key background characteristics; and (2) identify disparity measures that are programmatically relevant and easy to interpret.

## METHODS

### Data

Data are from the Demographic and Health Surveys (DHS) Program, an international survey platform supported by the U.S. Agency for International Development. The DHS are nationally representative household surveys that provide comparable, high-quality data on population and health in 90 countries.^10^ All women 15–49 years of age living in sampled households are eligible for the women's interview, which collects information on fertility, family planning, maternal and child health, and more. All DHS have a stratified two-stage cluster design that results in a sample that is generally representative at the national level, regional level (departments, states), and residence level (rural/urban). Because data are collected using a common methodology and common questionnaire, DHS data are comparable across time and place. Further details of DHS survey design and methodology are described elsewhere.^11^

DHS have collected detailed information on background characteristics since the program's inception in 1984. Around 1990, DHS began collecting information on housing conditions and household ownership of assets, which enabled computation of the household wealth index, an important dimension of disaggregation in our study. Our analysis was therefore restricted to countries with 2 or more DHS since 1990. As of September 2017, 213 surveys from 55 countries were eligible (Supplement 1). Among the 55 study countries, the average number of surveys since 1990 was 3.9 (range: 2–9) and the average interval between the earliest and latest survey was 16 years (range: 3–25 years). We used the DHS Application Programming Interface (API), available at http://api.dhsprogram.com/, to obtain estimates of met demand by background characteristics from the 213 surveys. Of note, all estimates in the DHS API were adjusted for sampling weights and calculated using standardized definitions, and, thus comparable.

This study analyzes levels, patterns, and trends in disparity for met demand among 213 DHS surveys from 55 countries.

### Measures

#### Met Demand by Background Characteristics

Met demand—demand for family planning met with modern methods—is defined as the proportion of women using modern contraceptives among women with demand for family planning. The indicator's denominator—women with demand for family planning—is comprised of women using any method of contraception and women with unmet need for family planning (i.e., sexually active women who report a desire to delay, space, or limit childbearing and also report no current contraceptive use). The numerator—women whose demand for family planning is met—is comprised of women who report current use of modern contraception. The main elements of met demand are: unmet need, contraceptive prevalence, and modern contraceptive prevalence.

Met demand is typically disaggregated by various background characteristics including age (5-year groups between 15 and 49 years), education (none, primary, and secondary or higher), household wealth quintile, residence (urban, rural), and subnational region/administrative unit. The administrative unit refers to subnational geographic regions that have planning authority and/or program implementation responsibility, such as states in Nigeria and counties in Kenya. These estimates are available through the DHS API. Disparity can present itself through other characteristics, including race, ethnicity, and religion. These data are available from DHS as well and should be monitored in countries with variation across such background characteristics, but we did not employ them in our analysis since they are highly dependent on country context.

Since some countries —typically in South Asia and the Middle East and North Africa—depart from the standard DHS methodology and interview only ever-married women, we limited our analysis to estimates among women in union (i.e., currently married or living with a partner) for the purpose of comparison across all available study countries. Because the denominator of met demand includes only women who have demand for family planning, unmarried women consist of a small fraction and excluding them from our calculations generally made no notable impact on the national-level estimate. Across the 213 study surveys, there was an average 1.4 percentage-point difference between met demand among all women and met demand among married women. In a handful of countries in Western and Southern Africa, the differences were larger, with the largest difference being 11.6 percentage points in Sierra Leone. Since women's access to family planning information and services can vary by union status, in a large subset of countries where all women are interviewed, we examined estimates of met demand and disparity by current union status (in union, not in union).

#### Disparity Measurement

Disparity in health has been studied extensively,^12^^–^^20^ and disparity trends are measured in various ways depending on study objectives. For example, some studies have examined **rates of change** across subgroups to explore whether various subpopulations have experienced similar trends in improvements to health indicators.^12^^,^^20^ Persistent disparity, however, can be observed with comparable improvement across subgroups. Further, since a baseline level for a more disadvantaged subgroup is generally lower, there is a larger margin of improvement that is mathematically and programmatically possible.^21^ **Relative difference**, another measure of disparity, has been also studied.^12^^,^^13^ The advantage of monitoring relative difference over time is that changes in the underlying rates between subgroups are already adjusted.^21^ Relative difference may, however, over- or under-emphasize disparity when levels across subgroups are relatively low or high, respectively. An additional challenge in using relative difference is selection of a reference group, since the measure can be sensitive to the choice.^21^ A more widely used approach is to assess trends of **absolute differences** across subgroups.^12^^,^^13^^,^^22^^–^^24^ Absolute difference is an intuitive summary measure of disparity. Its trend, however, is determined by various trends among subgroups. Decreasing disparity can result from different trends in 2 subgroups—for example, improvement in both groups but more rapid improvement in a disadvantaged group, or improvement in the disadvantaged group but no improvement or even deterioration in the advantaged group.^24^

While all disparity measures are informative, for the purpose of monitoring national-level disparity trends in addition to national-level averages, absolute difference is generally recommended.^12^ We therefore chose to use absolute differences to examine equality trends.

#### Reference Group Choice

For background characteristics with generally clear socioeconomic order, our reference group was the most advantaged. We calculated the percentage-point difference between the most- and least-advantaged groups—that is, between secondary or higher education and no education; highest and lowest household wealth quintile; urban and rural; and currently in union and not in union. If disparity patterns follow the usual socioeconomic pattern of health service utilization (i.e., the more advantaged, the higher the met demand), the absolute difference between the most- and least-advantaged socioeconomic subgroups would be the largest possible among all subgroups and greater than zero. Such patterns, however, do not necessarily exist in all disaggregates and subpopulations. We conducted preliminary analyses to compare absolute difference by typical socioeconomic order against the largest possible difference regardless of expected order (Supplement 2). Despite a few country-specific outliers, the two measures of socioeconomic disparity—the difference in levels between the usual least-advantaged socioeconomic category and the most-advantaged and the difference between the lowest and highest levels—correlated closely for education, household wealth, and residence. By union status, the pattern varied greatly.

Meanwhile, for two background characteristics—age and administrative unit—there was no straightforward way to order the subgroups across countries. We therefore calculated the absolute differences between the lowest and highest estimates among subgroups. The difference in these cases simply represented the magnitude of variation, which was always greater than zero.

In addition, the number of subgroups varied across the background characteristics, ranging from 2 (urban/rural and in union/not in union) to 7 (5-year age groups) or more, depending on survey design (administrative unit). We expect to see higher disparity when a population is disaggregated into more subgroups, given distribution of met demand in the population. Therefore, direct comparison of the disparity magnitude across different disaggregation dimensions is inappropriate.

### Analysis

Our unit of analysis was survey, and we conducted largely descriptive analyses. For any summary statistics, we used unweighted averages across countries regardless of their population size for two reasons. First, family planning policies and programs are generally developed and implemented at the national level. Second, the unit of SDG monitoring is expected to be the country, rather than an aggregated global average weighted by population size.

To understand disparity trends, we used a fixed-effect linear regression model to estimate an average annual absolute change per year, controlled for any unobserved country-level characteristics. We included 1 covariate—national-level estimate of met demand—in order to control for changes in the national-level average, which may be associated with the level of disparity. We used STATA 14.2 statistical software for all analyses (Stata Corporation, College Station, USA).

## RESULTS

### Levels and Trends of Disparity

**FIGURE 1:**
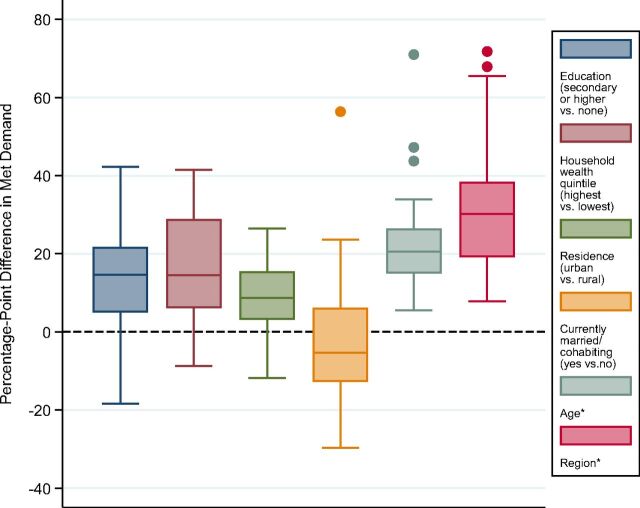
Boxplot Distribution of Within-Country Disparity in Family Planning Demand Met With Modern Methods (Percentage Point) Between the Most- and Least-Advantaged Subgroups,* by Background Characteristic All disparity measures are among women in union, except disparity by union status, which is among all women. Length of the box represents the interquartile range, the horizontal line in the box is median, the vertical line is a range of lower and upper adjacent values, and dots are outside values. Data were from the latest DHS from 55 countries. * Disparity by age or administrative unit represents the largest possible absolute gap among all subgroups.

Data from each country's latest survey showed sizeable within-country disparity (Figure 1). The median disparity in met demand by education was 15 percentage points (first box on the far left in Figure 1), by household wealth 15 percentage points (second box), and by residence (urban/rural) 9 percentage points (third box). Met demand among women in union was about 5 percentage points lower than among women not in union (median); however, the disparity pattern was not as consistent as disparity by education, household wealth, or residence. In 38% of study countries, met demand was higher among women in union than among women not in union. In terms of age, met demand was about 21 percentage points higher in the best-performing age group (often women ages 35–39, but not universally) compared with the lowest-performing age group (often women ages 15–19, though again not universally) (Supplement 3). Meanwhile, disparity by administrative unit/region presented the largest differentials, partly due to a large number of subgroups (average number of regions: 17, standard deviation [SD]=12, range: 3–54, n=55 latest surveys in each country). Specifically, the best-performing regions achieved, on average, 32 percentage points higher met demand than the poorest-performing regions (median: 30 percentage points) (Figure 1).

Data from each country's latest survey showed sizeable within-country disparity.

Turning to trends in magnitude of disparity, we observed that disparity by education, wealth, urban/rural residence, and age has decreased over time (bivariate model columns in the Table). For example, disparity by education has dropped by 0.55 percentage points each year. When controlling for changes in the national level of met demand, the magnitude of change in disparity by education, wealth, and urban/rural residence was reduced substantially (multivariate model columns in the Table). Nevertheless, we still observed statistically significant reductions in disparity by education, wealth, residence, and age (−0.24, −0.43, −0.19, and −0.36 percentage points per year, respectively). Meanwhile, we saw increases in disparity by region (0.36 percentage points per year) though this is likely a measurement artifact as DHS data have become more granular with larger sample sizes and more administrative units of estimation. There was no statistically significant change in disparity by union status in the multivariate model.

Disparity by education, wealth, urban/rural residence, and age has decreased over time.

**FIGURE 2:**
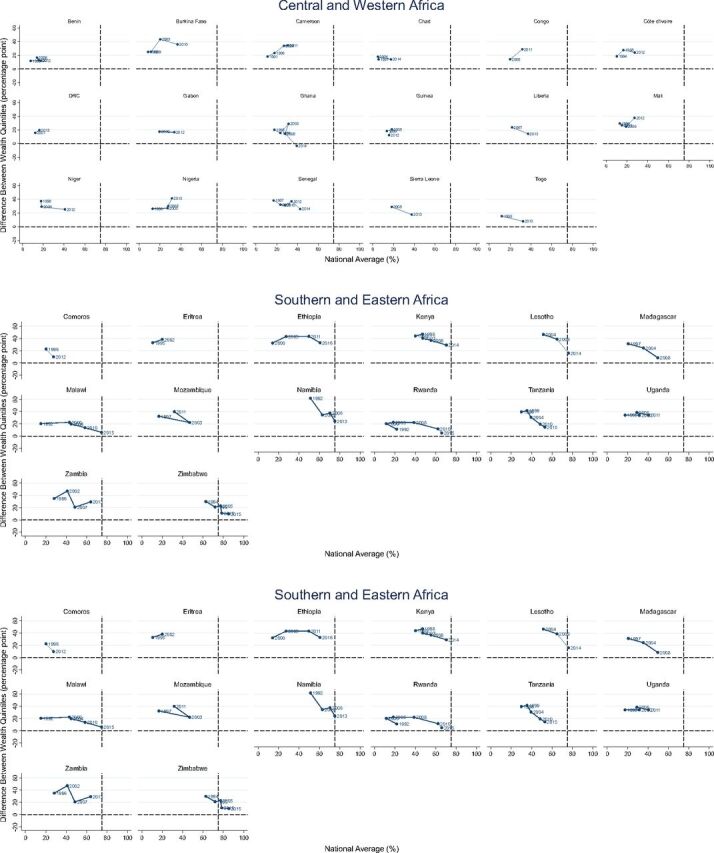
Trends in National-Level Family Planning Demand With Modern Methods (%) and Disparity by Wealth^a^ (Percentage Point), by Region and Country ^a^ Difference between the highest and lowest wealth quintiles.

Variation in disparity trends across countries needs to be noted. Figure 2 shows how the overall levels of met demand and disparity have changed by country over time. Each dot represents a survey, and a line is connected in the order of survey year. If a line moves from top left to bottom right (as shown in the example of Madagascar), it depicts that overall met demand has increased and disparity has decreased over time—as observed in most countries. However, a handful of countries, mostly in Central and Western Africa, had different patterns. For example, in Burkina Faso and Côte d'Ivoire, disparity decreased, following a period of a temporary increase, while the national-level met demand has increased continuously. Meanwhile, in Cameroon and Nigeria both disparity and the national level of met demand increased.

**TABLE.** Estimated Annual Changes in Disparity in Family Planning Demand Met With Modern Methods Between the Most- and Least-Advantaged Subgroups^a^ by Background Characteristic (Percentage-Point Change per Year)

Absolute Disparity by:	Bivariate Model		Multivariate Model^b^			
	Coeff. on Year	*P* Value	Coeff. on Year	*P* Value	Coeff. on National Average	*P* Value
Education (secondary or higher vs. none)	−0.55	<.001	−0.24	.007	−0.27	<.001
Household wealth (highest vs. lowest quintile)	−0.61	<.001	−0.43	<.001	−0.17	.003
Residential area (urban vs. rural area)	−0.42	<.001	−0.19	.002	−0.20	<.001
Union status (in union vs. not in union)	0.75	<.001	0.17	.21	0.48	<.001
Age^a^	−0.05	.46	−0.36	<.001	0.28	<.001
Administrative unit^a^	0.47	<.001	0.36	.001	0.10	.12

a Disparity by age or administrative unit represents the largest possible absolute gap among all sub-groups. All disparity measures are among women in union, except disparity by union status which is among all women.

b Multivariate models include 2 covariates: year and national average.

Note: Sample size for regression is 213 surveys from 55 countries, except disparity by union status where only 161 surveys from 45 countries were available for analysis.

Overall met demand has increased and disparity has decreased over time in most countries.

Even countries that meet the “at least 75% with met demand” benchmark have varying levels of disparity by wealth. In Morocco and Malawi, for example, no disparity by wealth was observed when the 75% benchmark was attained. On the other hand, large disparity still exists in Namibia and Lesotho even though both have reached the benchmark. And virtually zero disparity exists in Ghana even though it is still quite far from achieving the benchmark. Supplement 1 presents the national level of met demand and disparity by various disaggregates and survey.

**FIGURE 3:**
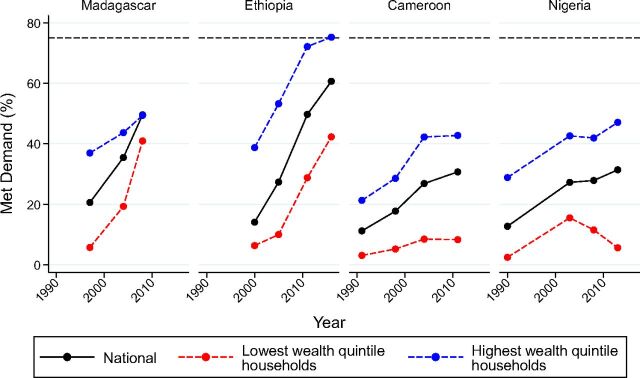
Illustrative Examples of Varying Trends of Family Planning Demand Met With Modern Methods (%), Nationally and by Household Wealth Quintile

Country context is further examined in Figure 3, which presents trends in disparity among 4 countries with increasing national levels of met demand—Madagascar, Ethiopia, Cameroon, and Nigeria. While disparity by wealth reduced substantially in Madagascar, it increased in Nigeria. In Ethiopia and Cameroon, the level of disparity between the highest and lowest wealth quintiles remained constant between the last 2 surveys for 2 different reasons: Met demand across wealth quintiles increased in Ethiopia but stagnated in Cameroon.

### Interpretation of and Correlation Among Measures by Various Disaggregates

**FIGURE 4:**
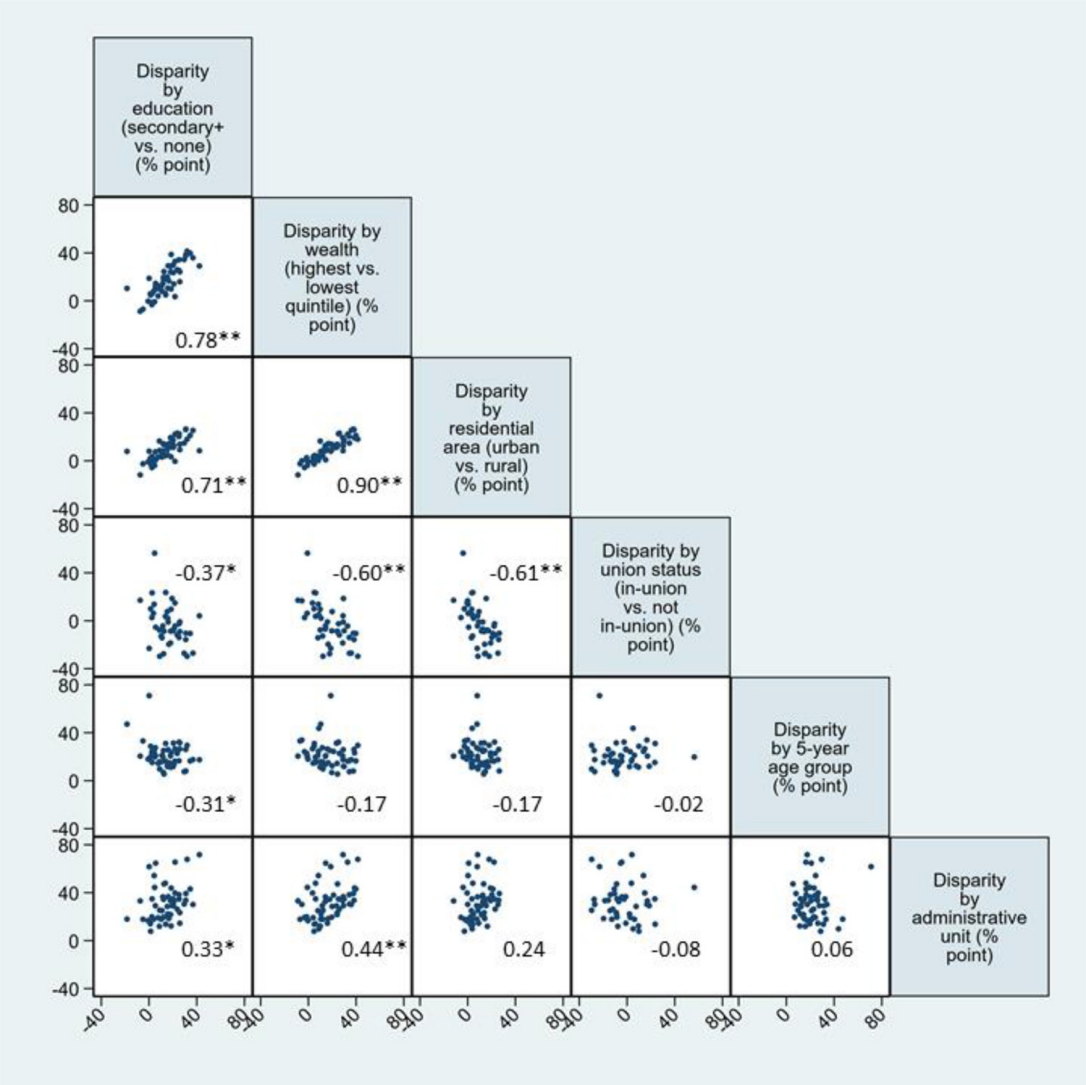
Correlation Matrix Among Various Disparities in Family Planning Demand Met With Modern Methods, With Correlation Coefficient * *P* value <.05; ** *P* value <.01. For education, household wealth, and residential area, disparity is between the most- and least-advantaged subgroups. For age and administrative unit, disparity represents the largest possible absolute gap among all subgroups. Data are from the latest DHS from 55 countries. All disparity measures are among women in union, except disparity by union status, which is among all women.

To inform our identification of a parsimonious set of disaggregates to monitor disparity in ways that are programmatically relevant and easy to interpret, we assessed correlation among disparity measures and distribution of population by the background characteristics. We found that there were statistically significant positive correlations among 4 disparity measures—education, wealth, residence, and administrative unit (Figure 4)—as expected since education, wealth, and residence are highly correlated. Pairwise correlation coefficients among disparity by education, wealth, and residence were especially high (0.90 by wealth and residence; 0.78 by education and wealth; and 0.71 by education and residence). Disparity by union status, however, was negatively related with disparity by education, wealth, and residence. This may be due to selective data availability, which were largely from sub-Saharan Africa and Latin America. Perplexingly, disparity by age was negatively associated with disparity by education and was not significantly associated with any other disparity measures.

There were statistically significant positive correlations among 4 disparity measures: education, wealth, residence, and administrative unit.

**FIGURE 5:**
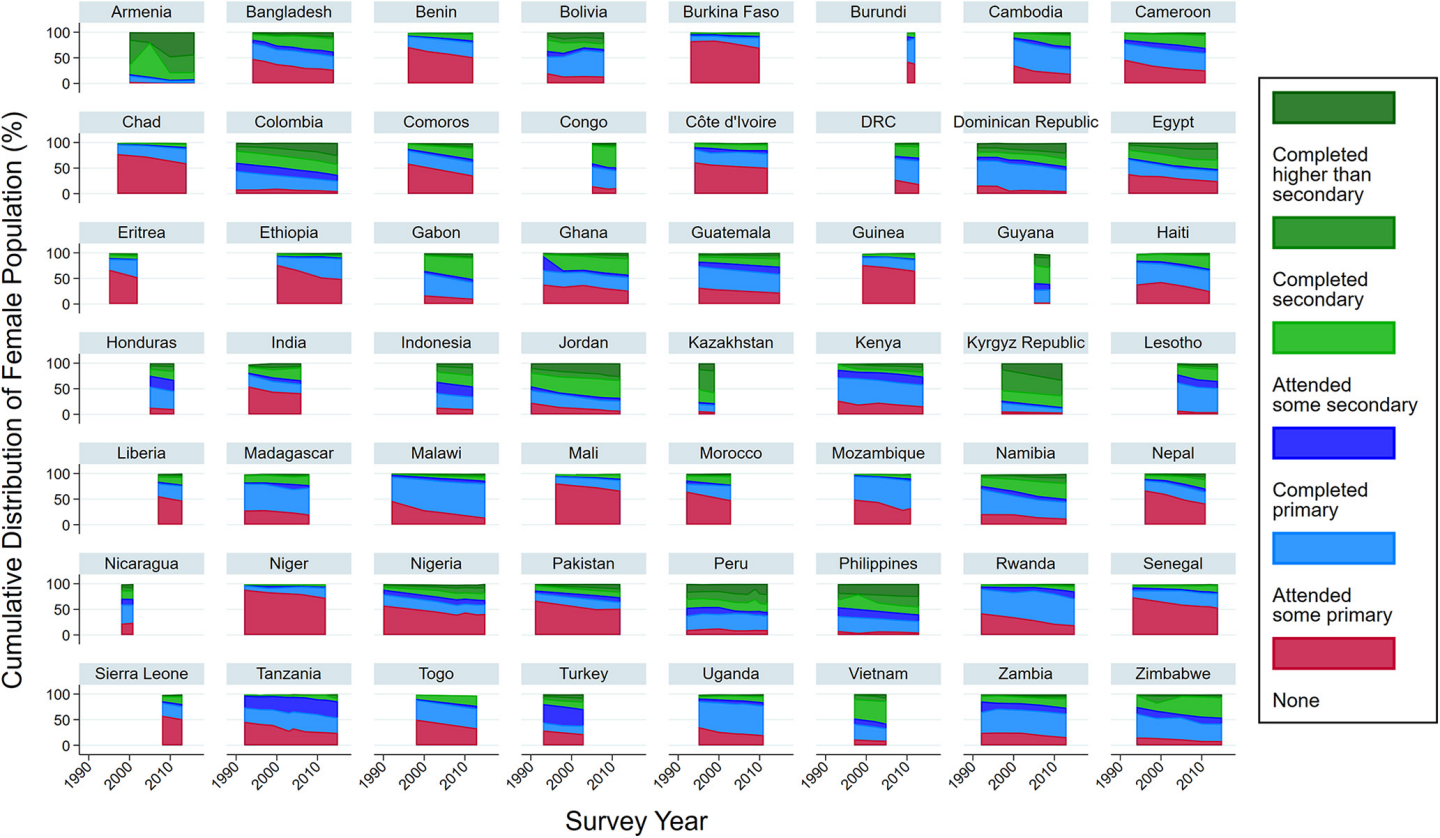
Changes in the Distribution of Educational Attainment Among Female Population 6 Years and Older Graph area varies by the number of surveys and intervals between them. Source: DHS Program Application Programming Interface (API), available at http://api.dhsprogram.com/.

Among the 4 correlated disparity measures, education had the greatest interpretation challenges in terms of comparability over time and across countries. Though improving overall, educational attainment showed great variation across countries (Figure 5). To elaborate, 2 countries can have the same level of disparity between the most- and least-educated women. But if they have vastly different levels and/or trends of female educational attainment, interpretation and implications of the disparity are different. Take, for example 2 countries with similar levels of met demand and similar levels of disparity by education—Niger and Cameroon. The percentage of women who attended secondary school or higher is still minimal in Niger, while it has grown steadily in Cameroon. The education categories have different percentages of women over time, which makes the scope, breadth, and depth of the disparity difficult to ascertain. Similar challenges present when observing disparity by urban/rural status with countries having different levels and rates of urbanization and also periodic changes in urban-rural designation based on censuses. Monitoring disparity by education and residence gives only magnitude of disparity between subpopulations of undefined size. It is therefore neither comparable nor easily interpretable between countries.

## DISCUSSION

There is substantial variation in the level of disparity in met demand for family planning across countries. As expected, the level of met demand largely follows socioeconomic order. In terms of trends, on average, disparity by socioeconomic characteristics has decreased over the last 2 decades, controlling for national-level increases. Nevertheless, while most countries in our analysis witnessed reduced disparity in met demand as the overall level of met demand increased, the level and magnitude of change is context specific. For SDG 3.7, meeting the benchmark of “at least 75%” does not necessarily imply that equality will be achieved. We also find that disparity between administrative units has increased significantly. This may be due to uneven progress among regions, but it is also due to the increasing number of administrative units for which a survey was designed to provide estimates.

While most countries witnessed reduced disparity in met demand as the overall level of met demand increased, the level and magnitude of change is context specific.

We now turn our attention to our second specific aim of this study: identifying key measures of disparity necessary for effective programming and monitoring at national and global levels. In this space, it's worth discussing the inconsistent disparity pattern by union status, which is noteworthy and reflects multiple factors: differential access to family planning by union status, differential strength of fertility intentions by union status, and/or potential data issues. Specifically, one would expect lower levels of met demand among unmarried women in countries where access to family planning services is limited to unmarried women due to restrictive requirements and/or provider bias.^25^^,^^26^ On the other hand, unmarried sexually active women may have more steadfast fertility intentions and therefore may be more likely to use contraception, resulting in higher met demand. Finally, there may be selection bias at 2 levels. Unmarried women who self-report to be sexually active may have certain characteristics that are associated with increased use of contraception or increased reporting of contraceptive use. Also, data are unavailable in countries where the cultural context precludes participation of never-married women in the women's interview. For these reasons, drawing conclusions, especially at the global level, about disparity by union status remains challenging.

Among the other dimensions of disaggregation included in our analysis—education, wealth, residence, age, and administrative unit—one indicator rises to the fore for monitoring within-country disparity at the global level: disparity by wealth quintile. Since relative wealth quintile distribution is constant, its interpretation is clear over time and across countries: the difference between the wealthiest 20% and poorest 20% of the population at any time and in any country. Additionally, since disparity by wealth is highly correlated with disparity by education, residence, and region, it can serve as a tracer indicator for disparity among other subpopulations; If disparity by wealth is high, it is likely that disparity by other characteristics is also high. Importantly, the measure has been also routinely used to monitor progress toward Millennium Development Goals.^27^ We recognize that the absolute level of poverty or wealth in a same quintile may vary substantially across countries and over time within a given country. Regardless, from the perspective of ensuring inclusive development, our interest is to monitor within-country equality at any given time—even with improving economy—which wealth quintiles allow.

If disparity by wealth is high, it is likely that disparity by other characteristics is also high.

Moving from the global to the national level, it becomes critical for an indicator of disparity to have programmatic relevance. Disparity by wealth can be programmatically meaningful at the country level when policies and programs are designed to target the poor, such as subsidized health care costs for low-income individuals and families. More often, however, intervention programs in a country are targeted to specific geographic areas, which often serve as proxies for reaching disadvantaged groups of individuals with certain background characteristics. For example, it can be challenging and inefficient to identify target households based on wealth quintile and implement interventions for them, so programs instead target regions with high poverty. Thus, the more programmatically relevant disparity dimension at the national level is administrative unit, where geographies in greatest need are easily identified and budgeting and planning can be determined. While monitoring disparity at the administrative level will inform policies and programming to reduce disparity, we also recognize the importance of monitoring disparity by other background characteristics, including urban/rural divides, that many country-level programs already use to direct limited resources to the most underserved.

At the national level, the more programmatically relevant disparity dimension is administrative unit, where geographies in greatest need are easily identified and budgeting and planning can be determined.

A consideration with this approach is potential comparability issues over time if a substantial change is made in the administrative systems. Further, in countries where planning and budgeting is determined at second or lower administrative units, there may be need to disaggregate by even lower administrative units. Because measurement of met demand requires population-level survey data,^28^^–^^30^ data availability at subregional levels is challenging even with improved routine health information systems. Considering technical challenges and limited financial and human resources to conduct large-scale surveys, subregional data may come from statistical model-based estimation,^28^^–^^30^ though it will require efforts to explain the data and limitations such as uncertainty of estimates, which may be larger than survey sampling error, to data users, or oversampling in selected regions where programmatic investment is concentrated. Another consideration with the approach we describe herein is that comparisons between highest and lowest groups mask variation among the intermediate groups, which may have very different spreads based on country context. Where possible, the spread of disparity among all subgroups should also be monitored. Relatedly, it is important to note that disparity by union status and age may also have additional programmatic relevance in countries where discrimination against unmarried and/or younger women is prevalent or perceived to be problematic. Finally, in using any measures of disparity, incorporating and communicating uncertainty in estimates by background characteristics is the next step to be explored.

## CONCLUSION

Using DHS data among 55 countries, we report a wide range of disparity in met demand across various dimensions. While disparity has decreased, our data show persistently high disparity even among countries with high national levels of met demand. To achieve the SDG promise that “no one is left behind,” within-country disparity needs to be monitored and addressed at global, national, and, where possible, subnational levels. At the global level, we recommend monitoring within-country disparity by wealth quintile, as it provides comparable and easily interpretable information. At the national level, we recommend monitoring by administrative unit, considering its programmatic relevance. Our recommendations are applicable to other health areas. Strong correlation among socioeconomic characteristics implies that disparity by wealth mirrors disparity by other socioeconomic characteristics for health areas beyond family planning.^12^^,^^18^^,^^27^ Additionally, the programmatic importance of data disaggregated by region applies to all health areas, including and beyond family planning.

## Supplementary Material

18-00012-Choi-Supplements.docx

## Notes

**Peer Reviewed**

**Funding:** None.
