# Supplementary material for: Monitoring Progress in Equality for the Sustainable Development Goals: A Case Study of Meeting Demand for Family Planning
Source: Glob Health Sci Pract. 2018 Jun 27;6(2):390–401. doi: 10.9745/GHSP-D-18-00012 (PMC6024626; doi:10.9745/GHSP-D-18-00012)
Supplement: 18-00012-Choi-Supplements.docx [file 18-00012-Choi-Supplements.docx]

**SUPPLEMENT 1.** List of 213 Demographic and Health Surveys From 55 Countries Analyzed, the National Average Met Demand for Family Planning, and Disparity by Various Disaggregates

| Country | **Survey**  **Year** | **Met Demand Among Women in Union (%), National Average** | **Absolute Difference in Met Demand Across Subgroups (percentage points)** | | | | | | |
| --- | --- | --- | --- | --- | --- | --- | --- | --- | --- |
|  |  |  | **Between the Most- and Least-Advantaged Socioeconomic Subgroups** | | | |  | **Between the Highest and Lowest Estimates Across Subgroups** | |
|  |  |  | **Education** | **Household Wealth** | **Residential Area** | **Union Status** |  | **Age** | **Subnational Unit** |
| Armenia | 2000 | 28.4 | - | 19.3 | 8.1 | - |  | 15.2 | 19.8 |
| Armenia | 2005 | 26.9 | - | 20.3 | 7.7 | - |  | 21.0 | 18.5 |
| Armenia | 2010 | 39.8 | - | 19.7 | 17.8 | - |  | 39.7 | 40.8 |
| Armenia | 2016 | 40.2 | - | 21.1 | 12.7 | - |  | 24.4 | 38.0 |
| Bangladesh | 1994 | 55.0 | 3.8 | 1.9 | 7.3 | - |  | 19.6 | 24.7 |
| Bangladesh | 1997 | 60.6 | 2.1 | 6.2 | 10.1 | - |  | 15.2 | 35.5 |
| Bangladesh | 2000 | 60.7 | 4.3 | 10.4 | 4.5 | - |  | 12.6 | 25.7 |
| Bangladesh | 2004 | 64.8 | -1.0 | 1.6 | 4.3 | - |  | 14.3 | 34.3 |
| Bangladesh | 2007 | 65.4 | 0.1 | 0.6 | 4.6 | - |  | 14.4 | 30.0 |
| Bangladesh | 2011 | 69.7 | 2.3 | -0.6 | 3.0 | - |  | 14.3 | 20.1 |
| Bangladesh | 2014 | 72.7 | 2.5 | -1.4 | 2.5 | - |  | 22.9 | 19.9 |
| Benin | 1996 | 7.7 | 14.2 | 11.9 | 7.0 | -9.8 |  | 7.1 | 5.9 |
| Benin | 2001 | 15.5 | 18.4 | 12.8 | 5.6 | -5.8 |  | 11.8 | 13.7 |
| Benin | 2006 | 13.8 | 14.4 | 16.7 | 6.8 | -21.7 |  | 9.9 | 12.1 |
| Benin | 2012 | 17.4 | 10.2 | 12.0 | 4.1 | -12.8 |  | 15.7 | 17.9 |
| Bolivia | 1994 | 24.0 | 32.7 | 49.8 | 22.8 | -4.3 |  | 12.9 | 33.9 |
| Bolivia | 1998 | 33.6 | 33.5 | 48.5 | 25.5 | -12.3 |  | 24.8 | 31.6 |
| Bolivia | 2003 | 43.0 | 27.2 | 31.9 | 16.9 | -0.3 |  | 11.6 | 38.6 |
| Bolivia | 2008 | 42.9 | 20.3 | 30.0 | 17.4 | -8.4 |  | 11.8 | 34.9 |
| Burkina Faso | 1993 | 8.5 | 37.6 | 24.9 | 24.4 | -33.1 |  | 6.1 | 28.3 |
| Burkina Faso | 1999 | 11.4 | 45.0 | 25.2 | 29.2 | -38.6 |  | 1.3 | 30.8 |
| Burkina Faso | 2003 | 20.2 | 49.1 | 43.4 | 37.0 | -41.9 |  | 7.3 | 45.5 |
| Burkina Faso | 2010 | 36.9 | 36.8 | 35.9 | 25.4 | -27.1 |  | 17.6 | 30.2 |
| Cambodia | 2000 | 33.1 | 8.2 | 20.7 | 7.5 | -13.1 |  | 23.6 | 34.2 |
| Cambodia | 2005 | 41.7 | 7.6 | 9.8 | 1.6 | - |  | 17.1 | 19.8 |
| Cambodia | 2010 | 51.8 | -7.7 | -6.9 | -7.1 | 20.9 |  | 20.7 | 29.7 |
| Cambodia | 2014 | 56.4 | -7.4 | -8.7 | -11.8 | 17.1 |  | 20.5 | 33.1 |
| Cameroon | 1991 | 11.2 | 13.9 | 18.2 | 6.6 | -5.9 |  | 26.7 | 16.0 |
| Cameroon | 1998 | 17.8 | 17.4 | 23.3 | 11.5 | -10.3 |  | 24.8 | 18.1 |
| Cameroon | 2004 | 26.9 | 32.4 | 33.8 | 18.5 | -27.9 |  | 13.0 | 41.0 |
| Cameroon | 2011 | 30.7 | 29.5 | 34.5 | 14.8 | -27.1 |  | 7.5 | 30.5 |
| Chad | 1997 | 5.6 | 21.1 | 14.1 | 11.6 | -10.3 |  | 2.7 | 17.5 |
| Chad | 2004 | 5.0 | 32.4 | 18.0 | 16.6 | -13.9 |  | 6.0 | 21.2 |
| Chad | 2014 | 17.5 | 20.8 | 14.1 | 12.7 | -6.9 |  | 13.5 | 32.6 |
| Colombia | 1990 | 68.4 | 14.2 | 27.1 | 10.1 | 0.4 |  | 22.2 | 7.4 |
| Colombia | 1995 | 70.9 | 13.7 | 25.2 | 11.1 | 4.7 |  | 27.0 | 5.3 |
| Colombia | 2000 | 73.6 | 12.7 | 16.7 | 10.2 | 4.7 |  | 23.8 | 6.1 |
| Colombia | 2005 | 78.6 | 10.4 | 14.4 | 4.8 | 5.5 |  | 22.1 | 28.2 |
| Colombia | 2010 | 83.7 | 11.0 | 9.1 | 2.7 | 2.8 |  | 22.1 | 34.8 |
| Colombia | 2015 | 86.3 | 13.7 | 6.0 | 4.3 | 3.7 |  | 16.4 | 47.9 |
| Comoros | 1996 | 20.1 | 8.0 | 22.6 | 5.7 | -19.2 |  | 23.4 | 10.8 |
| Comoros | 2012 | 27.5 | 8.7 | 10.0 | 16.5 | -7.7 |  | 15.8 | 12.0 |
| Congo | 2005 | 19.9 | 12.4 | 14.0 | 8.5 | -10.8 |  | 13.4 | 10.3 |
| Congo | 2011 | 31.7 | 18.0 | 28.8 | 19.3 | -18.5 |  | 20.7 | 33.4 |
| Côte d'Ivoire | 1994 | 10.3 | 14.9 | 18.6 | 9.6 | -27.2 |  | 5.1 | 12.0 |
| Côte d'Ivoire | 1998 | 16.6 | 20.4 | 27.7 | 12.3 | -16.3 |  | 11.6 | 14.3 |
| Côte d'Ivoire | 2012 | 27.6 | 18.1 | 24.1 | 11.5 | -8.7 |  | 14.4 | 21.8 |
| DRC | 2007 | 12.2 | 8.9 | 15.6 | 9.5 | -15.4 |  | 8.0 | 17.7 |
| DRC | 2013 | 16.2 | 12.1 | 19.6 | 13.8 | -8.4 |  | 6.8 | 19.6 |
| Dominican Rep. | 1991 | 68.2 | 19.3 | - | 10.2 | -9.9 |  | 62.4 | 23.6 |
| Dominican Rep. | 1996 | 75.9 | 7.1 | 13.5 | 4.8 | 14.2 |  | 45.6 | 17.9 |
| Dominican Rep. | 1999 | 77.2 | -7.2 | 7.6 | -5.0 | 4.0 |  | 55.2 | 19.3 |
| Dominican Rep. | 2002 | 80.0 | 0.3 | 9.8 | -0.7 | 15.2 |  | 40.2 | 11.8 |
| Dominican Rep. | 2007 | 83.3 | -1.3 | 4.0 | -2.9 | 15.2 |  | 33.5 | 15.8 |
| Dominican Rep. | 2013 | 83.0 | 1.4 | 4.8 | -2.8 | 10.0 |  | 28.7 | 7.8 |
| Egypt | 1992 | 64.0 | 17.2 | - | 16.0 | - |  | 35.7 | 35.2 |
| Egypt | 1995 | 66.8 | 13.5 | 28.8 | 14.1 | - |  | 25.6 | 33.7 |
| Egypt | 2000 | 77.2 | 11.2 | 16.8 | 6.8 | - |  | 15.2 | 22.3 |
| Egypt | 2003 | 78.8 | 7.5 | 14.1 | 7.4 | - |  | 14.6 | 21.6 |
| Egypt | 2005 | 79.0 | 7.4 | 8.8 | 3.3 | - |  | 10.8 | 21.1 |
| Egypt | 2008 | 80.1 | 4.8 | 11.2 | 5.8 | - |  | 18.9 | 18.6 |
| Egypt | 2014 | 80.0 | 1.1 | 5.9 | 2.1 | - |  | 19.6 | 14.1 |
| Eritrea | 1995 | 10.6 | 38.3 | 33.0 | 25.7 | -48.8 |  | 14.5 | 26.9 |
| Eritrea | 2002 | 20.0 | 30.8 | 38.3 | 26.0 | -15.7 |  | 26.1 | 33.7 |
| Ethiopia | 2000 | 14.1 | 35.8 | 32.4 | 38.9 | -31.2 |  | 9.2 | 43.3 |
| Ethiopia | 2005 | 27.4 | 42.8 | 43.2 | 41.8 | -32.0 |  | 11.0 | 48.5 |
| Ethiopia | 2011 | 49.7 | 32.3 | 43.4 | 29.5 | -14.8 |  | 17.8 | 63.9 |
| Ethiopia | 2016 | 60.7 | 21.8 | 32.9 | 22.1 | -4.4 |  | 16.4 | 65.5 |
| Gabon | 2000 | 19.5 | 11.7 | 17.6 | 11.9 | -16.9 |  | 3.4 | 10.1 |
| Gabon | 2012 | 33.7 | 16.6 | 16.6 | 14.2 | -19.6 |  | 15.0 | 18.5 |
| Ghana | 1993 | 17.7 | 30.1 | 20.2 | 10.9 | -30.3 |  | 9.7 | 16.3 |
| Ghana | 1998 | 23.5 | 8.8 | 15.3 | 8.3 | -1.5 |  | 12.0 | 19.0 |
| Ghana | 2003 | 31.3 | 16.7 | 29.0 | 15.3 | -5.3 |  | 25.8 | 23.1 |
| Ghana | 2008 | 28.0 | 9.8 | 14.3 | 5.5 | -9.4 |  | 26.1 | 25.9 |
| Ghana | 2014 | 39.2 | 2.4 | -3.2 | -5.6 | 2.7 |  | 24.2 | 19.4 |
| Guatemala | 1995 | 45.2 | 42.0 | 63.1 | 29.4 | -0.9 |  | 38.1 | 41.7 |
| Guatemala | 1999 | 47.5 | 33.9 | 58.0 | 20.3 | -9.2 |  | 30.5 | 33.4 |
| Guatemala | 2015 | 65.6 | 16.1 | 29.3 | 12.6 | -1.1 |  | 25.9 | 36.7 |
| Guinea | 1999 | 13.5 | 23.0 | 18.4 | 15.7 | -23.6 |  | 16.2 | 11.6 |
| Guinea | 2005 | 18.4 | 18.1 | 20.7 | 17.1 | -23.0 |  | 5.2 | 28.2 |
| Guinea | 2012 | 15.7 | 8.9 | 12.3 | 8.6 | -29.7 |  | 9.8 | 25.4 |
| Haiti | 1994 | 21.1 | 15.9 | 20.8 | 12.6 | -28.1 |  | 18.3 |  |
| Haiti | 2000 | 33.7 | 10.3 | 9.4 | -0.2 | -1.9 |  | 28.2 | 22.1 |
| Haiti | 2006 | 35.8 | 13.2 | 19.2 | 7.3 | 1.1 |  | 15.7 | 22.4 |
| Haiti | 2012 | 44.8 | 3.7 | -1.0 | 0.4 | 6.2 |  | 21.2 | 17.8 |
| Honduras | 2005 | 68.9 | 14.4 | 22.9 | 10.8 | -1.9 |  | 18.3 | 32.4 |
| Honduras | 2011 | 76.0 | 15.7 | 10.6 | 4.9 | 2.1 |  | 11.7 | 22.2 |
| India | 1993 | 59.8 | 4.3 | 22.3 | 9.1 | - |  | 77.7 | 49.7 |
| India | 1999 | 66.6 | 1.2 | 18.6 | 6.7 | - |  | 75.2 | 59.6 |
| India | 2006 | 69.1 | 0.1 | 18.8 | 8.0 | -23.0 |  | 71.0 | 61.8 |
| Indonesia | 1991 | 70.6 | 9.6 | - | 2.5 | - |  | 14.0 | 52.5 |
| Indonesia | 1994 | 74.4 | 10.7 | - | 1.2 | - |  | 17.5 | 39.2 |
| Indonesia | 1997 | 77.0 | 5.1 | 6.5 | -1.8 | - |  | 21.1 | 36.4 |
| Indonesia | 2003 | 77.1 | 9.0 | 6.2 | -2.0 | - |  | 20.1 | 38.3 |
| Indonesia | 2007 | 77.0 | 12.4 | 4.5 | -2.1 | - |  | 21.2 | 46.2 |
| Indonesia | 2012 | 79.0 | 4.7 | -0.8 | -3.8 | 56.4 |  | 19.8 | 44.4 |
| Jordan | 1990 | 40.5 | 8.9 | 24.5 | 16.5 | - |  | 37.6 | 17.0 |
| Jordan | 1997 | 51.9 | 13.2 | 20.3 | 10.4 | - |  | 26.6 | 10.0 |
| Jordan | 2002 | 58.3 | 5.3 | 17.3 | 8.5 | - |  | 31.2 | 11.3 |
| Jordan | 2007 | 59.1 | 1.5 | 10.8 | 7.1 | - |  | 19.8 | 18.7 |
| Jordan | 2009 | 57.8 | 19.3 | 11.2 | 6.4 | - |  | 18.6 | 20.6 |
| Jordan | 2012 | 58.0 | 7.6 | 4.1 | 3.1 | - |  | 14.6 | 19.1 |
| Kazakhstan | 1995 | 61.1 | - | 2.1 | -1.0 | 10.4 |  | 47.5 | 10.4 |
| Kazakhstan | 1999 | 67.7 | - | 8.8 | 3.8 | 5.1 |  | 43.7 | 9.8 |
| Kenya | 1993 | 40.1 | 29.6 | 43.9 | 19.2 | -27.5 |  | 44.1 | 30.6 |
| Kenya | 1998 | 47.0 | 30.2 | 47.0 | 14.8 | 4.2 |  | 40.2 | 38.8 |
| Kenya | 2003 | 47.2 | 44.0 | 40.3 | 15.7 | -3.3 |  | 31.3 | 69.4 |
| Kenya | 2008 | 55.4 | 38.7 | 36.7 | 11.4 | 2.7 |  | 25.5 | 59.7 |
| Kenya | 2014 | 70.5 | 42.2 | 29.1 | 8.4 | 4.1 |  | 17.5 | 71.8 |
| Kyrgyz Rep. | 1997 | 68.6 | - | 4.4 | 4.3 | - |  | 24.3 | 9.9 |
| Kyrgyz Rep. | 2012 | 62.1 | - | -5.4 | 0.0 | 16.7 |  | 34.0 | 30.3 |
| Lesotho | 2004 | 51.5 | 51.0 | 46.4 | 23.2 | -3.7 |  | 31.5 | 44.0 |
| Lesotho | 2009 | 64.9 | 24.4 | 38.7 | 18.8 | 1.9 |  | 24.3 | 27.9 |
| Lesotho | 2014 | 76.1 | 25.8 | 16.0 | 9.2 | -0.9 |  | 26.4 | 14.5 |
| Liberia | 2007 | 21.9 | 15.3 | 23.7 | 14.7 | -7.4 |  | 21.9 | 19.0 |
| Liberia | 2013 | 37.2 | 11.6 | 14.3 | 8.3 | -4.4 |  | 20.4 | 36.5 |
| Madagascar | 1992 | 10.4 | 16.2 | - | 18.0 | -0.4 |  | 13.2 | 11.6 |
| Madagascar | 1997 | 20.6 | 28.2 | 31.2 | 16.0 | 5.0 |  | 16.0 | 13.0 |
| Madagascar | 2004 | 35.5 | 26.8 | 24.4 | 11.1 | 4.2 |  | 11.3 | 18.1 |
| Madagascar | 2008 | 49.6 | 4.3 | 8.5 | 0.3 | 13.8 |  | 24.5 | 54.3 |
| Malawi | 1992 | 14.9 | 45.1 | 20.3 | 16.4 | - |  | 12.7 | 3.0 |
| Malawi | 2000 | 43.1 | 21.9 | 22.3 | 18.5 | 9.6 |  | 20.5 | 5.2 |
| Malawi | 2004 | 44.7 | 19.7 | 19.8 | 11.5 | 13.6 |  | 17.2 | 4.2 |
| Malawi | 2010 | 58.4 | 10.9 | 13.7 | 7.3 | 4.3 |  | 13.0 | 25.9 |
| Malawi | 2015 | 74.6 | 2.6 | 6.1 | 3.5 | 23.3 |  | 15.2 | 34.9 |
| Mali | 1996 | 13.2 | 41.4 | 29.4 | 20.0 | -18.2 |  | 9.8 | 26.7 |
| Mali | 2001 | 15.1 | 31.5 | 26.9 | 21.2 | -10.7 |  | 9.8 | 23.9 |
| Mali | 2006 | 19.3 | 22.3 | 24.8 | 16.8 | -12.4 |  | 10.3 | 23.5 |
| Mali | 2012 | 27.3 | 30.9 | 37.7 | 26.5 | -10.5 |  | 8.1 | 39.3 |
| Morocco | 1992 | 54.6 | 15.8 | 33.2 | 18.1 | - |  | 5.4 | 24.1 |
| Morocco | 2003 | 73.2 | -0.7 | -0.3 | -0.9 | - |  | 18.0 | 16.7 |
| Mozambique | 1997 | 16.7 | 40.9 | 32.2 | 27.1 | -7.6 |  | 22.7 | 48.8 |
| Mozambique | 2003 | 46.8 | 29.1 | 22.1 | 10.0 | -12.4 |  | 22.9 | 39.3 |
| Mozambique | 2011 | 31.8 | 34.5 | 39.8 | 21.2 | -10.6 |  | 16.5 | 43.1 |
| Namibia | 1992 | 51.3 | 34.4 | 61.7 | 39.8 | -28.0 |  | 27.2 | 48.5 |
| Namibia | 2000 | 63.0 | 22.9 | 34.5 | 14.5 | -4.5 |  | 13.4 | 57.0 |
| Namibia | 2006 | 70.4 | 29.1 | 37.6 | 17.7 | -12.6 |  | 22.3 | 27.3 |
| Namibia | 2013 | 75.1 | 25.8 | 24.2 | 13.8 | -9.0 |  | 31.6 | 23.4 |
| Nepal | 1996 | 42.7 | 9.4 | 33.6 | 22.8 | - |  | 63.5 | 9.7 |
| Nepal | 2001 | 52.8 | 5.7 | 31.5 | 21.7 | - |  | 51.9 | 8.5 |
| Nepal | 2006 | 60.8 | -14.1 | 20.7 | 8.6 | - |  | 50.2 | 18.6 |
| Nepal | 2011 | 56.0 | -18.4 | 10.4 | 7.8 | - |  | 47.2 | 18.0 |
| Nicaragua | 1998 | 73.4 | 16.3 | 23.7 | 12.3 | 4.4 |  | 22.0 | 30.4 |
| Nicaragua | 2001 | 79.4 | 15.3 | 19.9 | 9.5 | 7.6 |  | 12.4 | 28.5 |
| Niger | 1992 | 10.0 | 43.1 | - | 26.3 | -53.8 |  | 9.7 | 31.4 |
| Niger | 1998 | 17.8 | 44.9 | 37.5 | 31.6 | -40.3 |  | 13.6 | 31.7 |
| Niger | 2006 | 18.3 | 39.6 | 29.3 | 27.5 | -32.0 |  | 12.2 | 37.6 |
| Niger | 2012 | 40.8 | 25.2 | 25.3 | 22.5 | -2.5 |  | 32.2 | 34.7 |
| Nigeria | 1990 | 12.7 | 25.2 | 26.4 | 18.8 | -21.4 |  | 17.2 | 24.8 |
| Nigeria | 2003 | 27.2 | 27.3 | 27.1 | 15.3 | -15.6 |  | 10.6 | 32.3 |
| Nigeria | 2008 | 27.9 | 26.8 | 30.4 | 15.2 | -18.3 |  | 20.7 | 29.9 |
| Nigeria | 2013 | 31.4 | 31.9 | 41.5 | 18.0 | -29.7 |  | 29.5 | 67.9 |
| Pakistan | 1991 | 21.3 | 21.9 | 34.0 | 19.2 | - |  | 33.5 | 17.0 |
| Pakistan | 2006 | 39.6 | 9.7 | 23.3 | 12.4 | - |  | 34.6 | 13.0 |
| Pakistan | 2012 | 47.0 | 5.3 | 11.8 | 7.5 | - |  | 27.5 | 29.2 |
| Peru | 1992 | 40.7 | 34.6 | 47.3 | 28.5 | -4.2 |  | 29.6 | 35.6 |
| Peru | 1996 | 50.4 | 30.7 | 30.9 | 22.6 | -0.3 |  | 13.4 | 52.5 |
| Peru | 2000 | 60.5 | 20.6 | 23.9 | 16.8 | 9.9 |  | 13.3 | 51.5 |
| Peru | 2004 | 57.0 | 21.6 | 27.1 | 17.1 | -5.1 |  | 5.3 | 51.4 |
| Peru | 2007 | 57.4 | 16.0 | 25.1 | 15.0 | -9.8 |  | 11.8 | 51.8 |
| Peru | 2009 | 59.5 | 15.0 | 21.7 | 13.2 | -7.4 |  | 14.7 | 48.1 |
| Peru | 2010 | 59.6 | 11.4 | 23.0 | 13.7 | -8.2 |  | 13.1 | 50.9 |
| Peru | 2011 | 60.1 | 12.5 | 20.8 | 12.8 | -9.0 |  | 11.6 | 47.9 |
| Peru | 2012 | 61.1 | 12.6 | 24.5 | 13.4 | -8.7 |  | 5.5 | 47.4 |
| Philippines | 1993 | 35.5 | 23.7 | 22.9 | 7.5 | 3.0 |  | 25.4 | 32.7 |
| Philippines | 1998 | 39.0 | 21.4 | 16.6 | 8.0 | 9.2 |  | 21.5 | 36.2 |
| Philippines | 2003 | 46.8 | 23.2 | 15.5 | 1.5 | 30.9 |  | 27.0 | 45.6 |
| Philippines | 2008 | 46.8 | 29.8 | 9.5 | 2.0 | 20.3 |  | 26.0 | 41.4 |
| Philippines | 2013 | 51.8 | 21.8 | 3.5 | -0.4 | 14.9 |  | 26.6 | 39.8 |
| Rwanda | 1992 | 21.7 | 24.4 | 11.2 | 11.7 | -39.1 |  | 10.6 | 18.3 |
| Rwanda | 2000 | 11.5 | 23.7 | 20.1 | 18.3 | -10.6 |  | 7.8 | 19.9 |
| Rwanda | 2005 | 18.4 | 30.3 | 22.3 | 16.3 | 9.1 |  | 7.8 | 19.8 |
| Rwanda | 2008 | 38.7 | 30.2 | 22.1 | 12.9 | - |  | 25.6 | 18.5 |
| Rwanda | 2010 | 62.3 | 16.4 | 11.7 | 4.8 | 12.2 |  | 38.9 | 19.4 |
| Rwanda | 2015 | 65.9 | 14.0 | 5.0 | 4.3 | 23.6 |  | 31.1 | 13.7 |
| Senegal | 1993 | 13.2 | 37.3 | - | 19.7 | -41.6 |  | 17.6 | 16.5 |
| Senegal | 1997 | 16.9 | 40.3 | 38.4 | 28.0 | -53.5 |  | 19.9 | 19.6 |
| Senegal | 2005 | 23.5 | 34.3 | 32.3 | 20.7 | -24.4 |  | 18.8 | 36.1 |
| Senegal | 2010 | 28.0 | 25.5 | 31.3 | 20.6 | -1.5 |  | 22.0 | 29.1 |
| Senegal | 2012 | 34.3 | 20.6 | 37.2 | 27.6 | -6.2 |  | 35.7 | 26.6 |
| Senegal | 2014 | 42.5 | 23.6 | 26.0 | 23.0 | -11.9 |  | 22.8 | 26.3 |
| Sierra Leone | 2008 | 18.3 | 24.0 | 29.1 | 20.3 | -12.7 |  | 20.3 | 27.9 |
| Sierra Leone | 2013 | 37.5 | 12.2 | 17.8 | 14.2 | -27.6 |  | 25.3 | 34.4 |
| Tanzania | 1992 | 17.3 | 46.5 | - | 16.2 | -41.0 |  | 17.2 | 32.3 |
| Tanzania | 1996 | 30.0 | 29.2 | 39.5 | 25.5 | -2.8 |  | 17.7 | 39.6 |
| Tanzania | 1999 | 35.4 | 28.9 | 41.4 | 30.9 | -3.9 |  | 13.6 | 42.6 |
| Tanzania | 2004 | 39.4 | 38.6 | 30.5 | 23.3 | -10.0 |  | 18.6 | 47.7 |
| Tanzania | 2010 | 48.3 | 15.1 | 19.4 | 8.4 | -6.5 |  | 13.0 | 49.7 |
| Tanzania | 2015 | 52.9 | 5.6 | 14.5 | 0.8 | -5.4 |  | 20.9 | 64.7 |
| Togo | 1998 | 12.0 | 18.5 | 15.4 | 8.5 | -15.2 |  | 6.5 | 12.7 |
| Togo | 2013 | 32.3 | 10.9 | 8.0 | 3.1 | -14.2 |  | 21.2 | 25.0 |
| Turkey | 1993 | 44.7 | 26.6 | 28.9 | 13.8 | - |  | 31.7 | 11.7 |
| Turkey | 1998 | 48.4 | 22.3 | 26.0 | 10.9 | - |  | 28.2 | 15.5 |
| Turkey | 2003 | 54.8 | 20.5 | 23.5 | 11.2 | - |  | 31.4 | 29.1 |
| Uganda | 1995 | 17.4 | 30.5 | 34.1 | 32.9 | -21.2 |  | 11.6 | 20.6 |
| Uganda | 2000 | 31.5 | 40.1 | 34.0 | 33.0 | -22.1 |  | 10.6 | 27.9 |
| Uganda | 2006 | 29.0 | 30.7 | 38.5 | 27.9 | -29.5 |  | 16.2 | 42.8 |
| Uganda | 2011 | 40.4 | 25.1 | 34.3 | 20.3 | -13.9 |  | 16.6 | 38.4 |
| Vietnam | 1997 | 66.7 | 5.6 | 2.2 | -5.9 | - |  | 18.9 | 17.0 |
| Vietnam | 2002 | 66.6 | -5.0 | -6.9 | -2.5 | - |  | 33.2 | 17.8 |
| Yemen | 1991 | 12.8 | 37.9 | - | 23.6 | - |  | 14.9 | 9.1 |
| Yemen | 1997 | 16.1 | 19.0 | 31.2 | 19.8 | - |  | 17.5 | 10.9 |
| Yemen | 2013 | 46.9 | 18.6 | 38.7 | 19.1 | - |  | 23.2 | 43.9 |
| Zambia | 1992 | 19.7 | 35.1 | - | 21.4 | -27.4 |  | 14.4 | 28.5 |
| Zambia | 1996 | 28.2 | 32.6 | 35.1 | 21.3 | 0.3 |  | 8.3 | 32.9 |
| Zambia | 2002 | 41.0 | 37.5 | 47.1 | 28.6 | -2.7 |  | 19.0 | 31.7 |
| Zambia | 2007 | 48.5 | 18.3 | 20.7 | 16.4 | -2.1 |  | 11.7 | 38.3 |
| Zambia | 2013 | 63.9 | 19.3 | 29.4 | 15.5 | 18.6 |  | 12.1 | 29.2 |
| Zimbabwe | 1994 | 62.8 | 29.8 | 29.9 | 18.1 | -1.1 |  | 32.5 | 36.6 |
| Zimbabwe | 1999 | 71.8 | 24.6 | 21.1 | 15.5 | 3.8 |  | 30.3 | 28.6 |
| Zimbabwe | 2005 | 77.1 | 37.7 | 23.0 | 11.8 | 8.7 |  | 32.0 | 26.4 |
| Zimbabwe | 2010 | 78.4 | 8.3 | 11.1 | 3.8 | 7.7 |  | 17.6 | 20.0 |
| Zimbabwe | 2015 | 85.2 | 18.5 | 9.9 | 3.3 | 9.6 |  | 11.3 | 12.2 |

**SUPPLEMENT 2.** Comparison of Two Absolute Difference Measures by Background Characteristics: Difference Between the Highest and Lowest (Most- and Least-Advantaged) Subgroups vs. Largest Possible Differences Among Subgroups

Solid red line represents no difference between the two measures. Disparity by education, wealth, or residential area is among women in union.

Data from the latest DHS from each country (n=55). Disparity by union status includes the latest survey from 48 countries where all women regardless of marital status were interveiwed.

**SUPPLEMENT 3.** Age Pattern for Met Demand Among Women in Union: Predicted Value by 5-Year Age Group Using a Country-Level Fixed-Effect Regression Model

Data are from the latest DHS from 55 countries.
